# The changing incidence of Dengue Haemorrhagic Fever in Indonesia: a 45-year registry-based analysis

**DOI:** 10.1186/1471-2334-14-412

**Published:** 2014-07-26

**Authors:** Mulya Rahma Karyanti, Cuno S P M Uiterwaal, Rita Kusriastuti, Sri Rezeki Hadinegoro, Maroeska M Rovers, Hans Heesterbeek, Arno W Hoes, Patricia Bruijning-Verhagen

**Affiliations:** Department of Child Health, Division of Infection and Tropical Pediatrics, Cipto Mangunkusumo Hospital, Medical Faculty University of Indonesia, Jl. Diponegoro No.71, Jakarta, Indonesia; Julius Center for Health Sciences and Primary Care, University Medical Center Utrecht, Utrecht, The Netherlands; Department Ministry of Health Republic of Indonesia, Vector Borne Disease Control, Jakarta, Indonesia; Theoretical epidemiology, Faculty of Veterinary Medicine, University of Utrecht, Utrecht, The Netherlands

**Keywords:** Dengue haemorrhagic fever, Epidemiology, Incidence, Age, Indonesia

## Abstract

**Background:**

Increases in human population size, dengue vector-density and human mobility cause rapid spread of dengue virus in Indonesia. We investigated the changes in dengue haemorrhagic fever (DHF) incidence in Indonesia over a 45-year period and determined age-specific trends in annual DHF incidence.

**Methods:**

Using an on-going nationwide dengue surveillance program starting in 1968, we evaluated all DHF cases and related deaths longitudinally up to 2013. Population demographics were used to calculate annual incidence and case fatality ratios (CFRs). Age-specific data on DHF available from 1993 onwards were used to assess trends in DHF age-distribution. Time-dependency of DHF incidence and CFRs was assessed using the Cochrane-Armitage trend test.

**Results:**

The annual DHF incidence increased from 0.05/100,000 in 1968 to ~ 35-40/100,000 in 2013, with superimposed epidemics demonstrating a similar increasing trend with the highest epidemic occurring in 2010 (85.70/100,000; p < 0.01). The CFR declined from 41% in 1968 to 0.73% in 2013 (p < 0.01). Mean age of DHF cases increased during the observation period. Highest incidence of DHF was observed among children aged 5 to 14 years up to 1998, but declined thereafter (p < 0.01). In those aged 15 years or over, DHF incidence increased (p < 0.01) and surpassed that of 5 to 14 year olds from 1999 onwards.

**Conclusions:**

Incidence of DHF over the past 45 years in Indonesia increased rapidly with peak incidence shifting from young children to older age groups. The shifting age pattern should have consequences for targeted surveillance and prevention.

**Electronic supplementary material:**

The online version of this article (doi:10.1186/1471-2334-14-412) contains supplementary material, which is available to authorized users.

## Background

Dengue infection is the most rapidly spreading mosquito-borne viral disease in the world [1]. The World Health Organization (WHO) reported that the incidence increased dramatically over the last 50 years and that dengue virus infections expanded to new countries, and from urban to rural settings [1]. Approximately 2 · 5 billion people live in endemic countries of which about 1 · 8 billion (more than 70%) in Southeast Asia and the Western Pacific Region [1–4]. Annually, about 50 million dengue infections occur [2, 3], and approximately 500,000 patients are hospitalized because of dengue haemorrhagic fever (DHF), of whom a large proportion are children [2–7].

Demographic and societal changes such as population growth, urbanization, and modern transportation appear to play an important role in the increased incidence and geographical spread of dengue virus [8]. Furthermore, travellers from non-endemic countries to endemic dengue areas are at risk of contracting dengue disease, and pose a health threat to non-endemic regions where competent mosquito vectors are currently found [9–12].

Historically, DHF was predominantly observed in children. Over the past decades however, changes have been observed in the age-distribution of DHF cases in most countries both in Southeast Asia and Latin America [13–17]. Nowadays it is reported that a significant proportion of DHF cases occur among adolescent and adult patients in Southeast Asia [15, 17–19], and also in Latin American countries [20]. However, many studies describing shifting age patterns of DHF fail to report continuous observations over longer periods of time or report on specific locations and outbreaks [14–17].

Indonesia is one of the largest countries in the dengue endemic region, with a population of 251 million. The first 58 dengue cases in Indonesia were reported from Jakarta (DKI Jakarta) and Surabaya (East Java) in 1968 [21–24]. Since then increasing numbers of cases and geographical locations affected by dengue have been reported [21, 22, 25–30]. Dengue epidemiology in Indonesia has been described mostly in the form of case series, reporting on single outbreaks, or clinical and virological studies on DHF patients in confined geographical locations and selected years [31]. To date, there have been no comprehensive studies describing the incidence of dengue epidemiology over time in Indonesia and new data from recent years are lacking. Furthermore, data on age-specific dengue incidence in Indonesia are scarce, even though such information may have important implications for preventive measures. In only one study was the age distribution of DHF reported, showing that between 1975 and 1984 the median age of patients increased by 9 months [32]. The availability of the continuous nationwide Indonesian dengue surveillance registry, enabled us to describe the evolution of DHF incidence and case-fatality rates over a period spanning 45 years and to evaluate age-specific trends over time.

## Methods

### Surveillance system and case definition

In 1968, dengue became a notifiable disease in Indonesia [21, 22], and was included in the national disease surveillance system run by the Communicable Disease Center of the Indonesian Ministry of Health. This means that all suspected DHF cases presenting to healthcare facilities or hospitals must be evaluated within 24 hours by healthcare providers and reported to the district health authority, while awaiting serological confirmation. This is followed by epidemiological investigation and a vector control program according to National guidelines when indicated based on serologic, virologic or epidemiological confirmation of dengue [33, 34]. In addition, the dengue surveillance included state government and WHO training programs for medical officers in diagnosis and case management from 1968 onwards [32].

Since its inception, the national surveillance-training program applies the same WHO dengue classification system from 1968 [32], which classifies symptomatic dengue into dengue fever (DF) and DHF. Although several changes have been made in the WHO Dengue classification since 1968 [32] these have not been adopted in the Indonesian national surveillance system, such that definitions and criteria for reporting have remained stable over the entire observation period, apart from a minor change in dengue serology testing where the haemagglutination inhibition test was replaced by rapid diagnostic tests for serologic IgM and IgG dengue that were available in the field. DHF is defined as having at least the first two of the following four clinical manifestations: 1) sudden onset acute fever of 2 to 7 days duration, 2) spontaneous haemorrhagic manifestations or a positive Tourniquet test, 3) hepatomegaly, and 4) circulatory failure, in combination with haematological criteria of thrombocytopenia (= < 100.000 cells/mm3) and an = > 20% increased haematocrit. Dengue shock syndrome is defined as DHF plus a rapid, weak pulse with narrow pulse pressure or hypotension with cold, clammy skin, and restlessness [29, 32, 34–36].

### Case ascertainment

Every suspected case of DHF based on clinical and haematological criteria requires further investigation to support the diagnosis of dengue. DHF is classified as probable when additional supportive dengue serology from a single blood specimen is available or when there is an epidemiological link to a confirmed dengue case. Supportive dengue serology is defined as positive anti-DENV IgM in acute or convalescent serum sample and/ or a fourfold increase in IgG between the acute and the convalescent samples. DHF cases are classified as confirmed through virus isolation, or detection of viral antigen or RNA in serum [32–34]. This classification has continually been used nationwide by all govermental and private hospitals. Of all initially identified, suspected cases of DHF, only probable and confirmed cases are subsequently reported to the Communicable Disease Center of the Indonesian Ministry of Health by district health authorities and captured in the surveillance database. This database covers all 33 Indonesian provinces. From 1993 onwards data collected in the surveillance database on DHF cases also included the following age categories: less than 1 year, 1-4 years, 5-14 years, and older than 15 years [23, 32, 35, 37].

Annual geographical mapping of Indonesian provincial incidence rates of DHF was available for the years 2010-2013 and is included.

### Population

Population demographic data for 1968 to 2013 were based on civil registration records of village authorities [38], and obtained from the official national census data, the Central Bureau of Statistics in Indonesia.

### Statistical analysis

The DHF incidence by year and age group was calculated by dividing the number of new DHF cases identified from surveillance data by size of the population at risk. Dengue case fatality ratios were determined by the number of deaths from DHF divided by the number of DHF cases. Trends in annual incidence and case fatality ratios were analysed using Cochrane-Armitage trend tests [39].

Subgroup analyses were performed to study trends according to age group. In an attempt to rule out that any observed change of incidence is partly due to increasing awareness and reporting in the early stage of the registry, the most recent period (1986-2013) was analyzed separately. The Health Research Ethics Committee Medical Faculty University of Indonesia Cipto Mangunkusumo Hospital approved the study.

## Results

**Figure 1:**
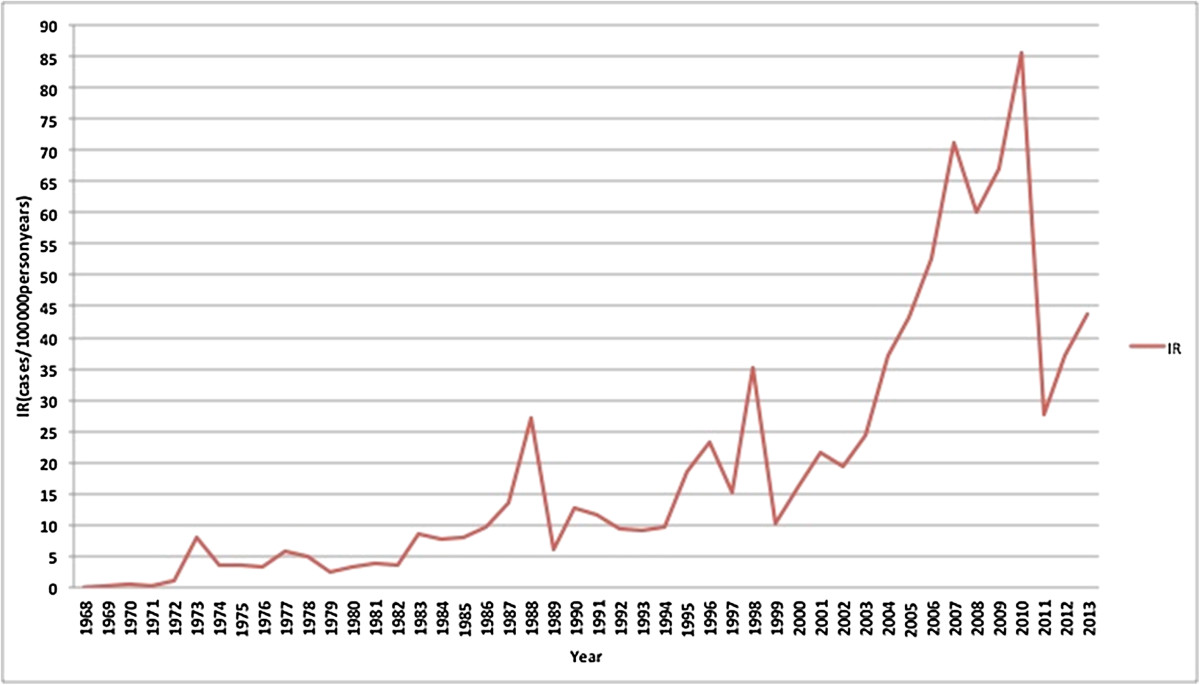
**Trends in incidence rate of DHF cases in Indonesia from 1968 to 2013, measured in numbers of cases per 100,000 person years.**

**Figure 2:**
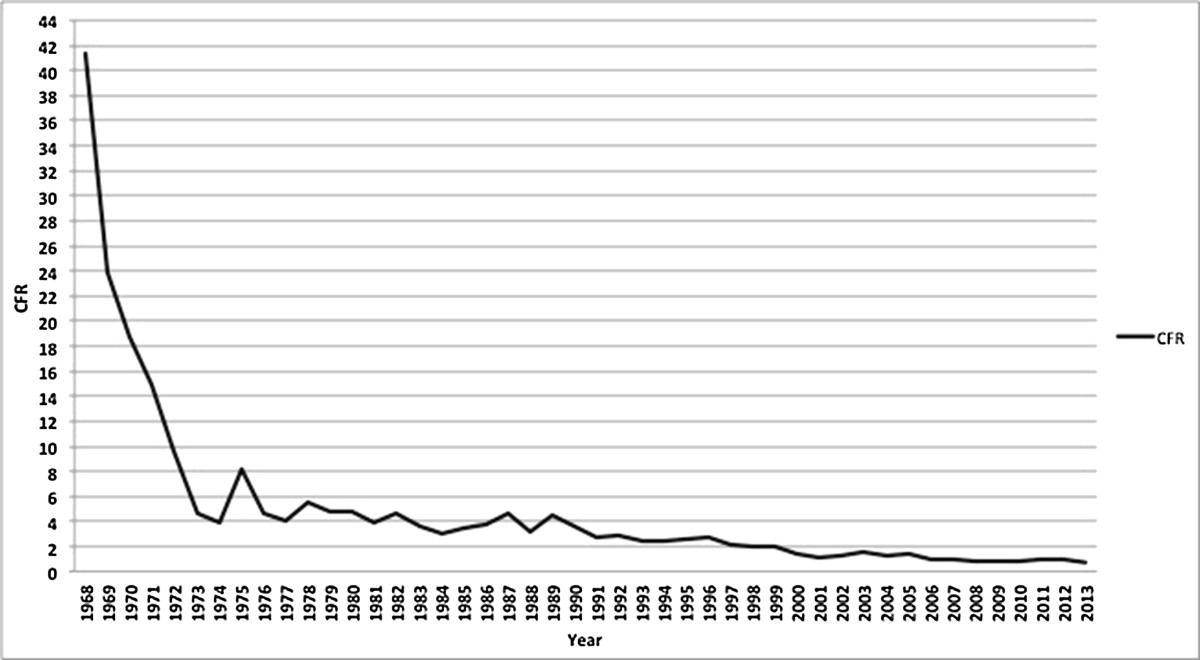
**Case fatality ratios of DHF cases in Indonesia from 1968 to 2013.**

Overall annual DHF incidence increased significantly from 0.05/100,000 in 1968 to to ~ 35-40/100,000 in 2013 in 2010 (p-value trend test: < 0.001). Superimposed epidemic peaks occurred with irregular intervals and a progressive increase in intensity. The highest epidemic peak was observed in 2010 with 86 DHF cases per 100,000 person-years. Figure 1 shows the incidence of DHF since 1968. Outbreaks were observed in the years 1973, 1988, 1998, 2007, and 2010. By contrast, the DHF case fatality ratio decreased from 41% in 1968 to 0.73% in 2013 (Figure 2, p-value for trend-test < 0.001) (Additional file 1).

**Figure 3:**
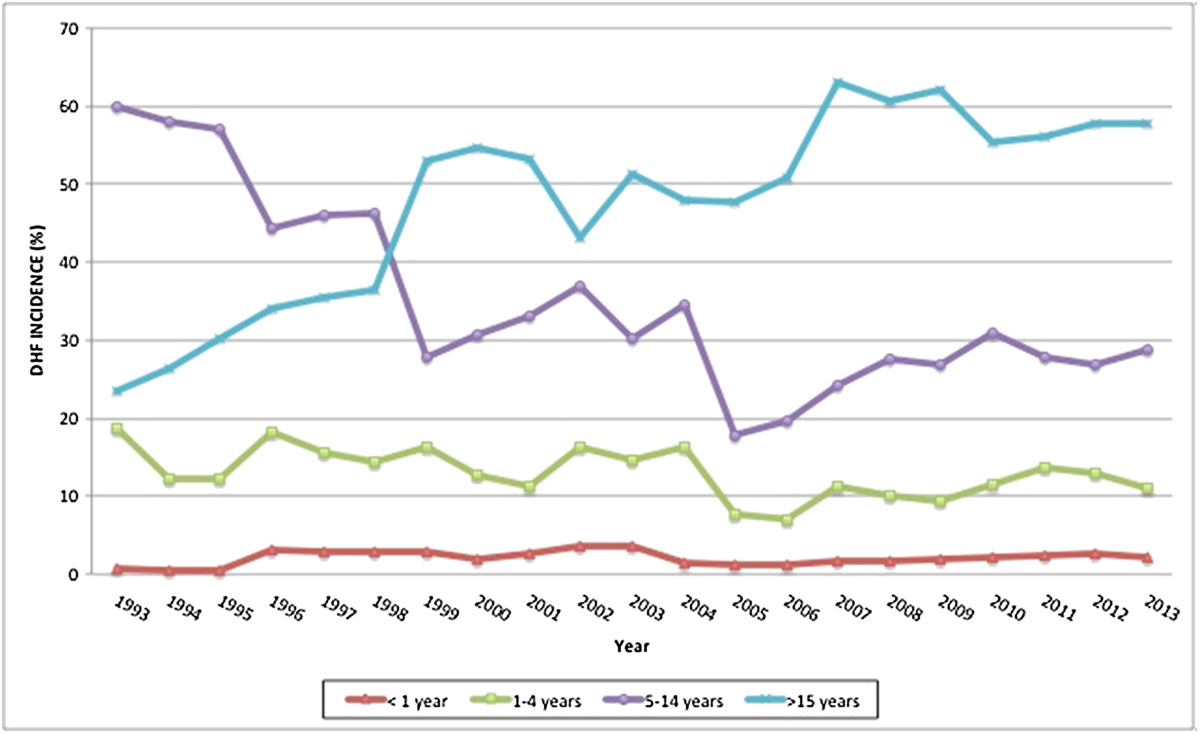
**Incidence (%) of DHF in the different age groups from 1993 to 2013.**

**Figure 4:**
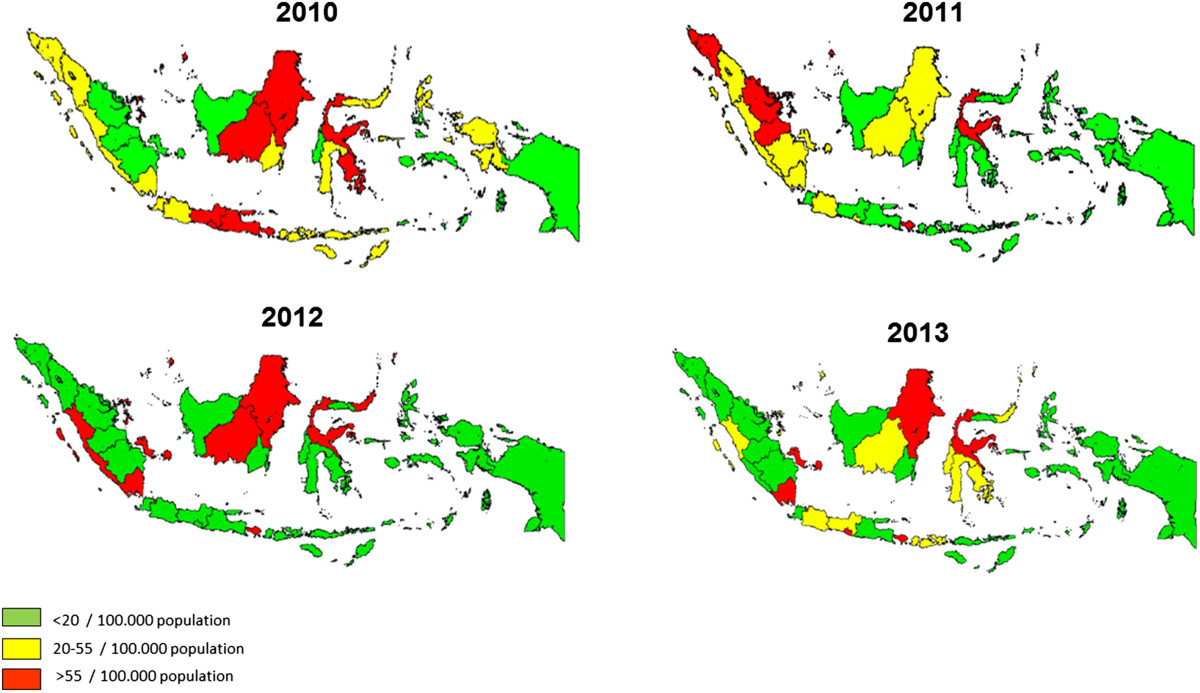
**Geographical mapping of Indonesian provincial incidence rates of DHF in 2010-2013.**

A separate analysis of the more recent 1986 to 2013 data revealed results compatible with the total period. The annual incidence of DHF increased significantly over time (p-value for trend-test < 0.01), while the case fatality ratio of DHF decreased considerably (p-value for trend-test < 0.01).From 1993 onwards, when age categories were also being recorded, the highest initial annual DHF incidence was observed in 5 to 14 year olds, but it steadily decreased from then on (p-value for trend-test < 0.01, Figure 3). In contrast, while the DHF incidence in 1993 in those aged over 15 years was much lower than in 5 to 14 year olds, a steady increase (p-value for trend-test < 0.01) in this age category was observed and the incidence surpassed that of young children around the year 1999. Throughout this period, the incidence in children aged less than 5 years was relatively low and remained stable.The geographical mapping of rates of DHF in Indonesian provinces over the years 2010-2013 is shown in Figure 4. Bali and DKI Jakarta had the highest incidence of DHF. In 2013, the five highest provincial incidences were observed in Bali (168.5/100,000), DKI Jakarta (104.0/100,000), DI Yogyakarta (96.0/100,000), East Kalimantan (92.7/100,000) and Sulawesi Tenggara (66.8/100,000). The geographical distribution did not change substantially over 2010-2013.

## Discussion

Our results showed that in the last 45 years, the DHF incidence increased rapidly in Indonesia with a pattern of intermittent hyperendemic years. The case fatality ratio, however, decreased over the same period. Based on age-stratified data available from 1993 onwards, there appears to be an important shift in the age of affected individuals; a steady decline in DHF incidence was observed over the years for children aged 5 to 14 years (the age group with highest DHF incidence historically), while the incidence in those aged over 15 years steadily increased and surpassed the decreasing incidence in younger children since 1999.

The strengths of our study are that annual DHF incidence and case fatality ratios have been documented continuously for 45 years, using the same WHO case definition and case classification of dengue, based on both clinical and laboratory diagnostic criteria without any substantial modification throughout the surveillance period. Furthermore, the data offered the opportunity to study trends by age groups.

Some potential limitations should also be discussed. Firstly, specific incidences according to DHF disease severity grade could not be reported since such information was not available from the reports studied. In addition, cases of severe dengue disease can present with atypical clinical manifestations such as massive hemorrhage and organ failure, neurologic disease, myocardiopathy, hepatic and renal failure, and may therefore not be reported as DHF, a problem also recognized in the WHO 2009 guidelines [40]. Secondly, mild DHF cases not presenting to healthcare facilities will not have been captured by the surveillance. Therefore, our findings likely reflect more severe symptomatic DHF cases requiring medical attention. Thirdly, cases of suspected DHF without serological testing performed do not end up in the surveillance database and this will have resulted in some underreporting.

In the early years of the surveillance program, limited communication and logistic facilities in several parts of Indonesia may have hampered DHF reporting to some extent. From the initial stage of surveillance onwards, the DHF training program will have gradually increased awareness among healthcare providers. We cannot completely rule out that the observed change of incidence is partly due to such increasing awareness and reporting. However, our sub-analysis of the most recent 20 years, clearly confirms our overall findings, thus rendering increased reporting an unlikely alternative explanation.

Increases in DHF incidence may be explained by increased vector density or abundance because of lack of effective mosquito control [41, 42], by increased human mobility [41, 42], and by altered virus-host interaction leading to increased infectivity and therefore more secondary infections [41]. The population in Indonesia is growing fast with an increase of almost 25% (from 206 to 251 million) between 2000 and 2013. Demographic and societal changes such as population density, urbanization and modern transportation probably contributed substantially to the increased incidence and geographical spread of dengue in Indonesia [31]. Over the most recent years of registry, there is a clear annual geographical distribution of DHF incidence with concentrations mainly in high-density populated areas. Possibly for that reason, this distribution does not seem to change much over time.

The DHF incidence in Indonesia has been increasing in over 15 year olds, while in the under 5 year olds it remained relatively low and stable, a pattern that has been observed in other high endemic Southeast Asia countries [6–8, 13, 15, 16, 43–46]. Demographic changes, i.e. changes in birth and death rates, may induce changes in the age distribution of cases and the periodicity of incidence [15]. Lower birth rate decreases the flow of susceptible individuals into the population and lower infant mortality increases the longevity of immune individuals [15]. Older age groups in endemic areas will be exposed to secondary dengue infection which mostly manifests as DHF, rather than the primary dengue infections that are predominantly found in younger age groups [4, 47]. Both births and infant mortality indeed decreased in Indonesia, i.e. from 22 births and 38 deaths per 1000 inhabitants in 2003 to 17 births and 26 deaths per 1000 inhabitants in 2013 and family size decreased to less than 3 children per family in 2010 [48]. These demographic changes will most likely have contributed to the upward shifting of age for DHF cases in Indonesia.

Epidemic outbreaks of DHF occurring every 8–10 years have also been reported in other countries, and might be the result of cross-protective immunity [43]. Adams et al [24], showed that DENV-4 was responsible for this epidemic pattern in Thailand which has an immunological cross-reaction with DENV-1 and, possibly, with other serotypes [43].

In Southeast Asia countries where dengue is endemic, only Malaysia and Singapore have active surveillance systems. Dengue surveillance programs in other Southeast Asia countries, including Indonesia, remains largely passive [17, 21, 32]. and it is estimated that incidence rates of dengue cases, based on reports from passive surveillance systems, are underestimated [42].

Further professional upgrading of dengue surveillance in Indonesia should be considered, including registration of more than only probable and confirmed DHF and including the expanded dengue syndromes with unusual manifestations according to the revised 2011 WHO South-East Asia Region Office (SEARO) dengue guidelines [49]. Extending the registry to include more detailed demographic data and information on social economic status, and disease severity will likely contribute to our further understanding of dengue epidemiology. Secondly, we believe that awareness about the shifting age-pattern is essential for clinical and public health vigilance and for the efficiency of preventive strategies. Education to create public awareness about the clinical signs of dengue in the adolescent age group and when to seek professional healthcare could improve timely medical interventions. Furthermore, vector-control programs should not only be aimed at houses, but also at schools and working areas [34].

## Conclusion

The incidence of DHF has increased substantially over the past 45 years, with superimposed epidemic outbreaks and requires re-enforcement of current surveillance practices. In contrast, the case fatality ratio clearly decreased during the same period. The shifting age pattern towards older age groups (above 15 years of age) should have consequences for targeted prevention strategies.

## Electronic supplementary material

Additional file 1: A supplementary table of incidence and CFR of DHF.(DOCX 37 KB)

Below are the links to the authors’ original submitted files for images.

Authors’ original file for figure 1

Authors’ original file for figure 2

Authors’ original file for figure 3

Authors’ original file for figure 4

## Abbreviations

DF: Dengue fever
DHF: Dengue haemorrhagic fever
DSS: Dengue shock syndrome
CFR: Case fatality rate
WHO: World Health Organization
IgM: Immunoglobulin M
IgG: Immunoglobulin G
DEN-V: Dengue virus
RNA: Ribonucleic acid
SEARO: Southeast Asia Region Office.
