# Supplementary material for: The changing incidence of Dengue Haemorrhagic Fever in Indonesia: a 45-year registry-based analysis
Source: BMC Infect Dis. 2014 Jul 26;14:412. doi: 10.1186/1471-2334-14-412 (PMC4122763; doi:10.1186/1471-2334-14-412)
Supplement: Supplementary file 1 — Additional file 1: A supplementary table of incidence and CFR of DHF.(DOCX 37 KB) [file 12879_2014_3713_MOESM1_ESM.docx]

A supplementary table of incidence and CFR of DHF

| Year | IR (cases /100000personyears) | CFR (%) |
| --- | --- | --- |
| 1968 | 0.05 | 41.3 |
| 1969 | 0.14 | 23.9 |
| 1970 | 0.4 | 18.8 |
| 1971 | 0.22 | 14.9 |
| 1972 | 1.14 | 9.6 |
| 1973 | 8.14 | 4.6 |
| 1974 | 3.57 | 3.9 |
| 1975 | 3.47 | 8.1 |
| 1976 | 3.38 | 4.7 |
| 1977 | 5.69 | 4.1 |
| 1978 | 4.96 | 5.5 |
| 1979 | 2.37 | 4.8 |
| 1980 | 3.39 | 4.8 |
| 1981 | 3.96 | 3.9 |
| 1982 | 3.53 | 4.7 |
| 1983 | 8.65 | 3.6 |
| 1984 | 7.86 | 3 |
| 1985 | 8.14 | 3.4 |
| 1986 | 9.79 | 3.7 |
| 1987 | 13.5 | 4.6 |
| 1988 | 27.09 | 3.2 |
| 1989 | 6.09 | 4.5 |
| 1990 | 12.7 | 3.6 |
| 1991 | 11.56 | 2.7 |
| 1992 | 9.45 | 2.9 |
| 1993 | 9.17 | 2.40 |
| 1994 | 9.72 | 2.51 |
| 1995 | 18.50 | 2.52 |
| 1996 | 23.22 | 2.71 |
| 1997 | 15.28 | 2.22 |
| 1998 | 35.19 | 1.96 |
| 1999 | 10.17 | 2.00 |
| 2000 | 15.99 | 1.41 |
| 2001 | 21.66 | 1.08 |
| 2002 | 19.24 | 1.32 |
| 2003 | 24.30 | 1.55 |
| 2004 | 37.01 | 1.20 |
| 2005 | 43.31 | 1.36 |
| 2006 | 52.48 | 1.04 |
| 2007 | 71.18 | 1.00 |
| 2008 | 60.02 | 0.86 |
| 2009 | 67.00 | 0.86 |
| 2010 | 85.70 | 0.87 |
| 2011 | 27.67 | 0.91 |
| 2012 | 37.11 | 0.90 |
| 2013 | 43.01 | 0.73 |
