# Supplementary figures and images for: The changing incidence of Dengue Haemorrhagic Fever in Indonesia: a 45-year registry-based analysis
Source: BMC Infect Dis. 2014 Jul 26;14:412. doi: 10.1186/1471-2334-14-412 (PMC4122763; doi:10.1186/1471-2334-14-412)

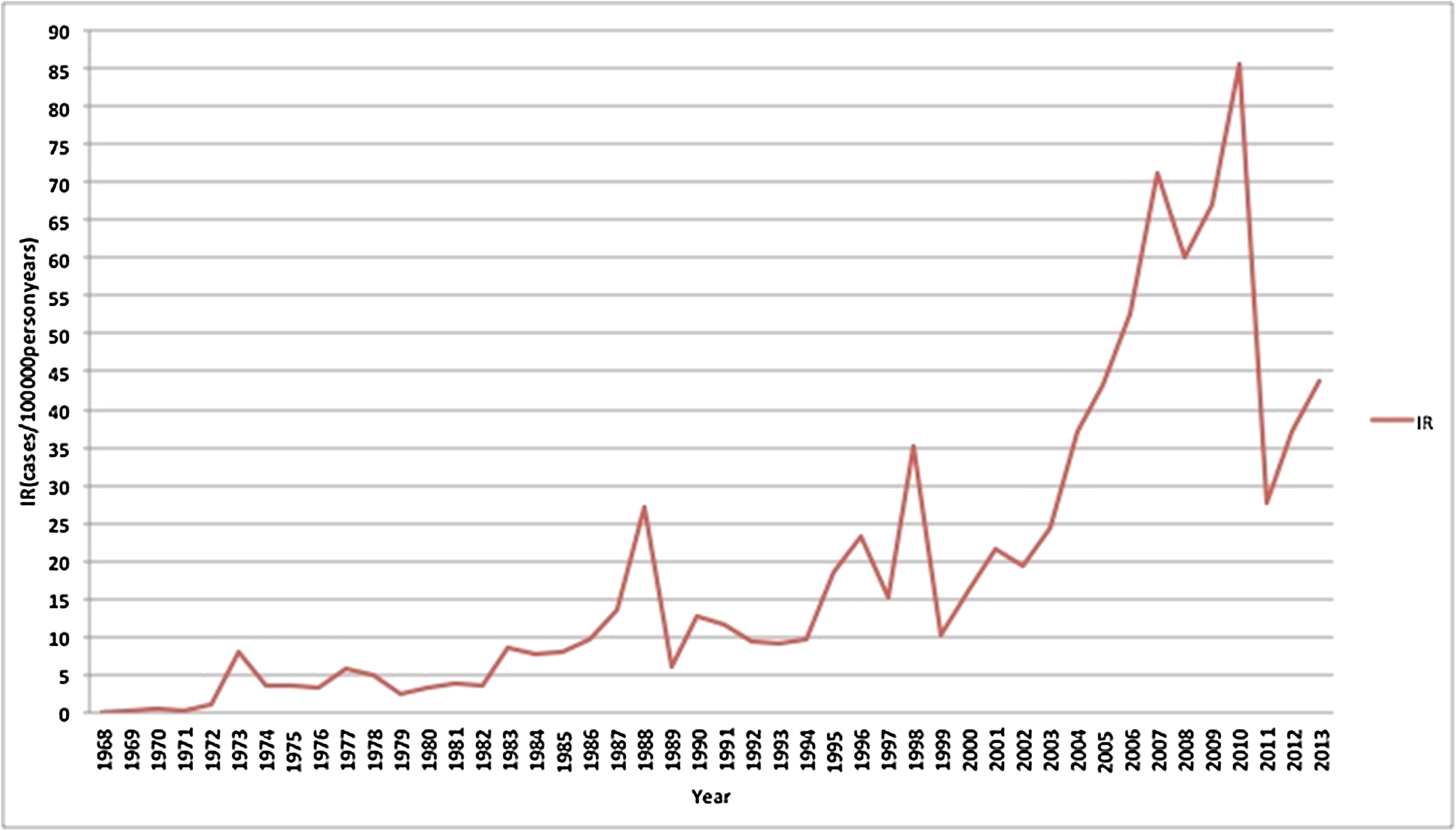

Supplement: Supplementary file 2 — Authors’ original file for figure 1 [file 12879_2014_3713_MOESM2_ESM.tif]

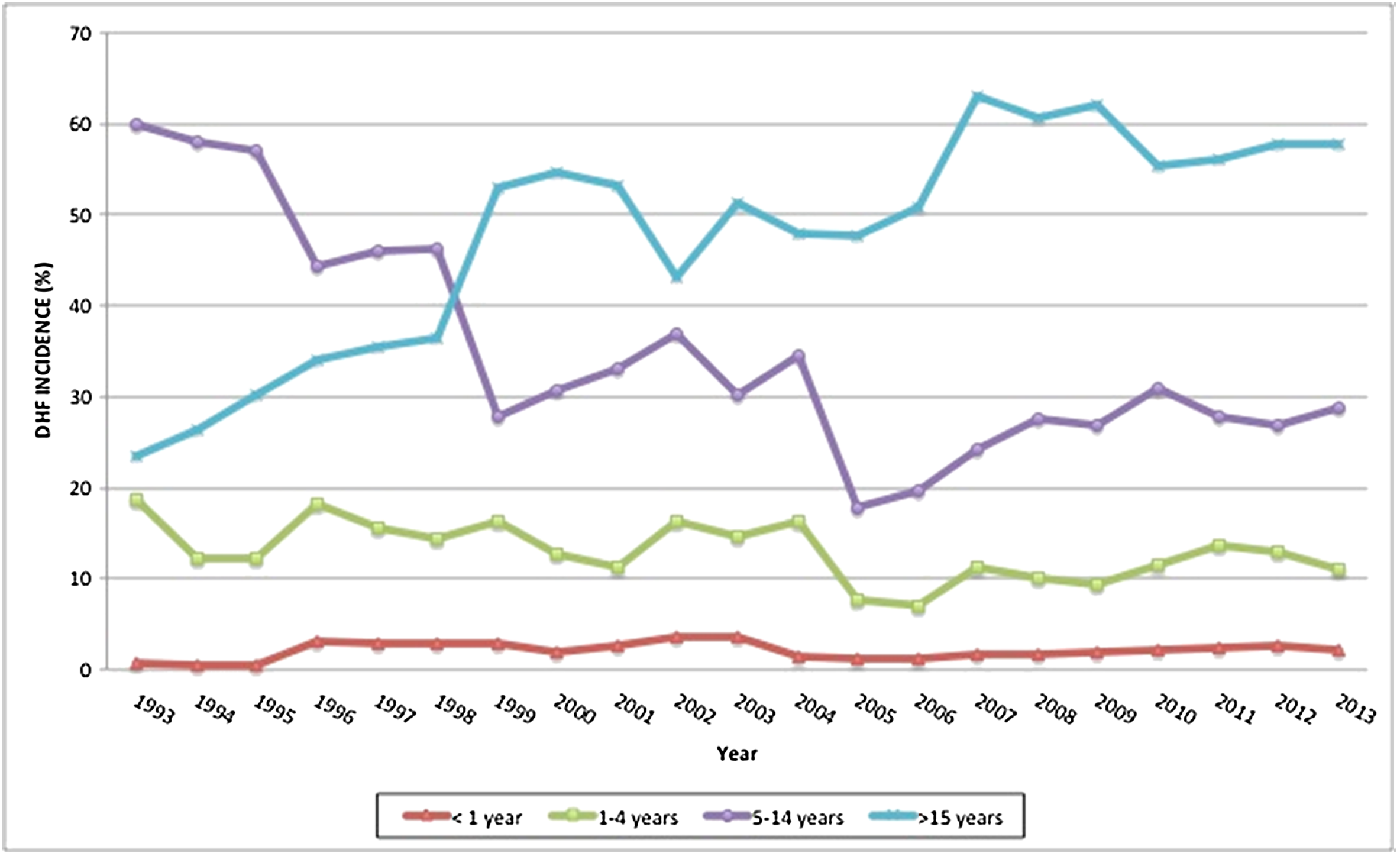

Supplement: Supplementary file 4 — Authors’ original file for figure 3 [file 12879_2014_3713_MOESM4_ESM.tif]

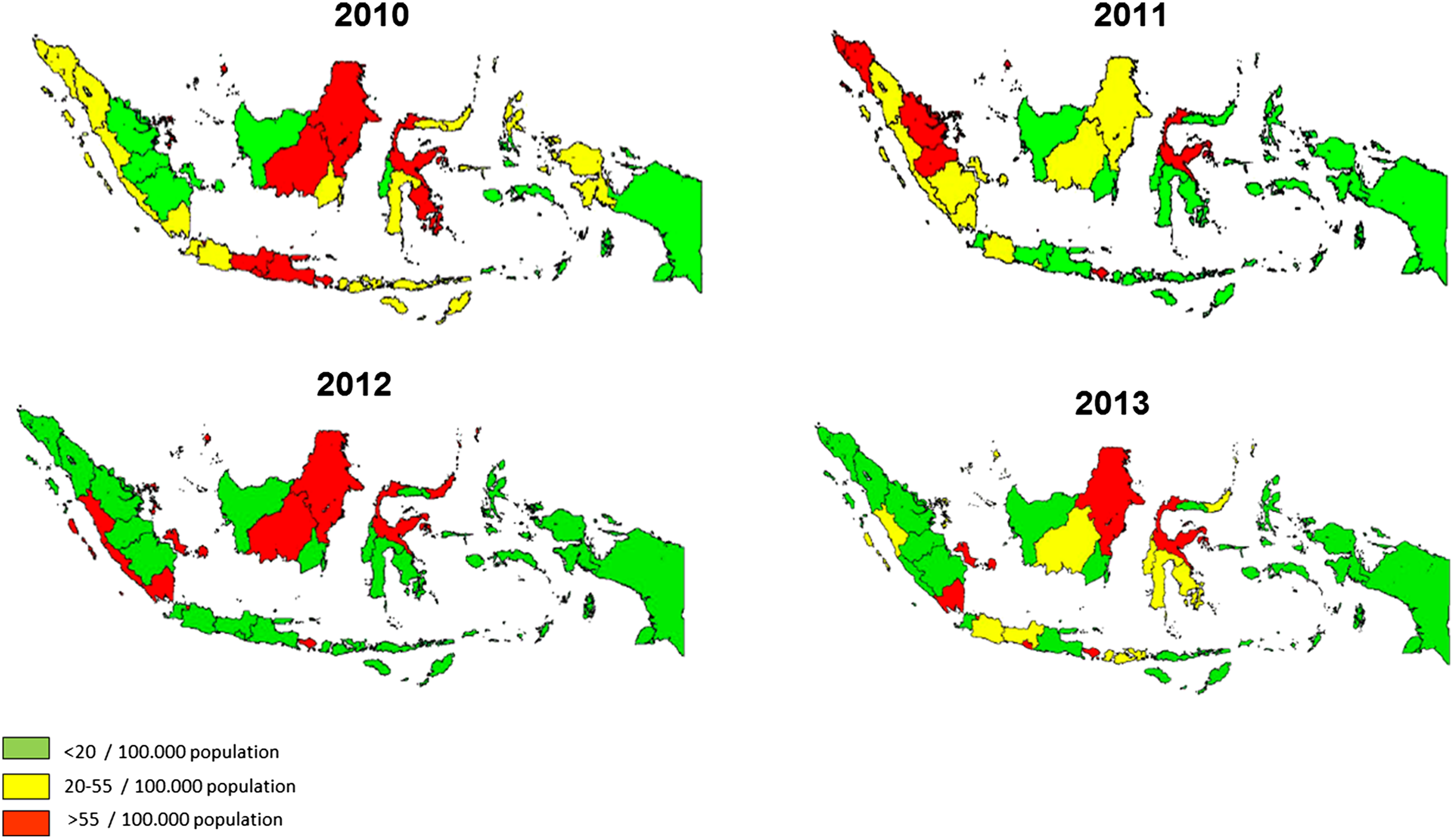

Supplement: Supplementary file 5 — Authors’ original file for figure 4 [file 12879_2014_3713_MOESM5_ESM.tif]
